# Prognostic signature and clonality pattern of recurrently mutated genes in inactive chronic lymphocytic leukemia

**DOI:** 10.1038/bcj.2015.65

**Published:** 2015-08-28

**Authors:** A M Hurtado, T-H Chen-Liang, B Przychodzen, C Hamedi, J Muñoz-Ballester, B Dienes, M D García-Malo, A I Antón, F de Arriba, R Teruel-Montoya, F J Ortuño, V Vicente, J P Maciejewski, A Jerez

**Affiliations:** 1 Hematology and Medical Oncology Department, Hospital Morales Meseguer, IMIB, Murcia, Spain; 2 Traslational Hematology and Oncology Research, Cleveland Clinic, Cleveland, OH, USA

## Abstract

An increasing numbers of patients are being diagnosed with asymptomatic early-stage chronic lymphocytic leukemia (CLL), with no treatment indication at baseline. We applied a high-throughput deep-targeted analysis, especially designed for covering widely TP53 and ATM genes, in 180 patients with inactive disease at diagnosis, to test the independent prognostic value of CLL somatic recurrent mutations. We found that 40/180 patients harbored at least one acquired variant with *ATM* (*n*=17, 9.4%), *NOTCH1* (*n*=14, 7.7%), *TP53* (*n*=14, 7.7%) and *SF3B1* (*n*=10, 5.5%) as most prevalent mutated genes. Harboring one ‘sub-Sanger' *TP53* mutation granted an independent 3.5-fold increase of probability of needing treatment. Those patients with a double-hit *ATM* lesion (mutation+11q deletion) had the shorter median time to first treatment (17 months). We found that a genomic variable: *TP53* mutations, most of them under the sensitivity of conventional techniques; a cell phenotypic factor: CD38-positive expression; and a classical marker as β2-microglobulin, remained as the unique independent predictors of outcome. The high-throughput determination of *TP53* status, particularly in this set of patients frequently lacking high-risk chromosomal aberrations, emerges as a key step, not only for prediction modeling, but also for exploring mutation-specific therapeutic approaches and minimal residual disease monitoring.

## Introduction

Nowadays, an increasing numbers of patients are being diagnosed with early-stage chronic lymphocytic leukemia (CLL), likely owing to the use of routine blood tests for health screening and the widespread availability of flow cytometry.^1, 2, 3^ Among this subset of CLL patients, most with a non-active disease and no treatment indication at baseline, different prognostic modeling approaches, incorporating traditional (clinical and laboratory), cytogenetic, immunophenotypic and immunoglobulin heavy-chain variable region gen (*IgVH*) status, have been proposed.^4, 5^

The contemporary vision of neoplasm development is based on a consecutive acquisition of genetic changes with a selection and expansion of the more fit population.^6, 7^ The heterogeneous course of CLL is, to a certain extent, driven by the diverse combinations of clones with acquired chromosomal lesions and somatic mutations.^8, 9^ Whole-exome studies (WES) have shown that the number of somatic variants per case is lower in CLL than that of those described in solid tumors and other leukemias, and that the set of genes affected is discreet.^10, 11, 12^ These two aspects make this disease a suitable candidate for deep-targeted sequencing, a technique focused on distinct genomic sites, which also enables reliable detection of subclonal mutations because of to its higher depth of coverage compared with WES. Using this more affordable and rapid strategy, recent studies have determined the prognostic impact of *TP53* subclones.^13^ In addition, the status of *ATM*, *NOTCH1* and *SF3B1*, also recurrently mutated in CLL, have been associated with impaired overall and treatment-free survival.^14, 15, 16^ Nevertheless, a comprehensive high-throughput sequencing study of these variants, assessing their clinical relevance, in the context of both traditional and newer factors, is lacking.

In this study, we propose to refine and apply a method for high-throughput targeted analysis of somatic recurrent mutations in CLL, especially designed for covering widely *TP53* and *ATM* genes. Our main aim is to assess the independency of the prognostic value of those variants, related to time to first treatment and survival, in patients with CLL and no indication for therapy at diagnosis.

## Materials and methods

### Patients

**Figure 1:**
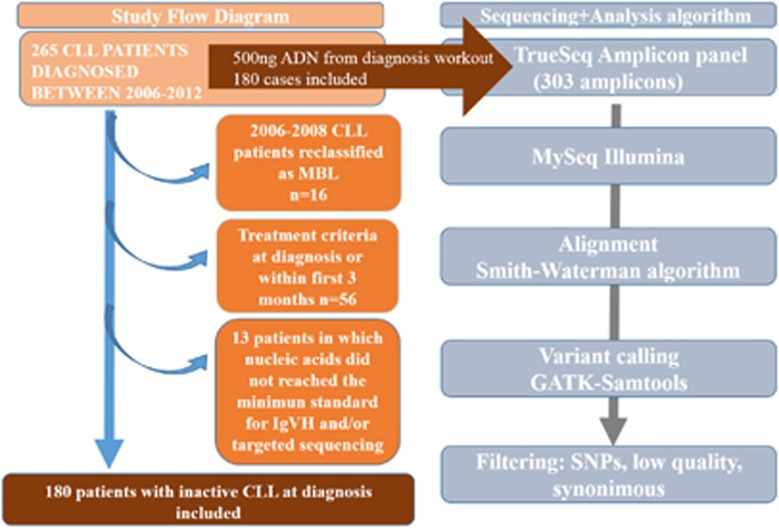
Study flow diagram. Visual representation of the exclusion criteria (left) and the targeted sequencing process pipeline (right).

From 2006 to 2012, presentation bone marrow aspirates or blood samples DNA was collected during the diagnostic workout from 265 consecutive CLL patients, after informed consent, according to the protocols approved by the Institutional Review Board of Hospital Morales Meseguer (EST-32/13) and with the Declaration of Helsinki. Patients who met criteria for an active disease at baseline, did not reach a minimum treatment-free follow-up of 3 months, or nucleic acids did not pass the quality control for either *IgVH* status or targeted sequencing, were excluded (Figure 1). Diagnosis and definition of active disease, requiring therapy, were achieved according to the International Workshop on Chronic Lymphocytic Leukemia established criteria.^17^ Time-to-first-treatment (TTFT) was measured from diagnosis to date of first treatment. Regular follow-up consisted of blood cell counts and clinical examinations every 3 months the first year after diagnosis, and henceforth, visits were carried out from 3 to 6 months, depending on patient risk.

### Diagnostic workout

Every patient underwent a flow cytometry characterization with a panel including CD45, pan B-cell markers (CD19, CD20, CD22, CD79b, and surface immunoglobulin light chains), markers for differential diagnosis with other B-cell chronic lymphoproliferative diseases (CD5, CD23, FMC7, CD10, CD81, CD103, CD25 and CD11c) and prognosis markers (CD38 and ZAP70) (Antibodies from BD Biosciences, San Jose, CA, USA).

Fluorescence *in situ* hybridization (FISH) analysis was performed on interphase nuclei at diagnosis from directly harvested peripheral blood or bone marrow samples according to the manufacturer's protocol and using the following commercially available probes (Abbott Molecular, Des Plaines, IL, USA): LSI MYB (6q23), LSI P53 (17p13.1)/ LSI ATM (11q22.3), LSI D13S319 (13q14.3)/CEP12, as reported.^18^ A minimum of 400 nuclei were scored for each probe or probe combination.

Immunoglobulin heavy-chain variable diversity (D)-joining (J) rearrangements were amplified from either reverse-transcribed total RNA (preferred source) or genomic DNA. Purified amplicons were sequenced either directly or on subcloning.^19^ Sequences were aligned to the ImMunoGeneTics for computation of mutational load.^21^ Sequences were considered mutated or not using the cutoff of 2% mismatch.^22^

### Targeted sequencing

We designed a TruSeq Custom Amplicon panel (Illumina, Inc. San Diego, CA, USA) containing 13 genes and covering 28.099 bases (Table 1). For some genes known mutation hotspots were targeted; and for those with a widespread localization of the lesions, the entire coding sequence was analyzed. The average amplicon size was 238 base pairs and ~99.1% of the regions were covered on both strands. Library preparation was performed according to manufacturer's instruction. Paired-end sequencing (2 × 250 bp) was performed with MiSeq v2.2 chemistry, and a mean depth of 938 reads/base within the regions of interest was obtained. Raw data were analyzed with IlluminaonJboard Real Time Analysis (RTA v.2.4.60.8) software and MiSeq Reporter.

### Variant call requirements and validation

The following conditions were established for a variant to be called: (i) to be non-synonymous; (ii) not to be listed in dbSNP database (NCBI Human Build 141); and (iii) a cutoff for any nucleotide position of 30 or more variant reads and a Q score >30 (see Variant call requirements: technique accuracy in Results). The filtered variant lists were manually reviewed and BAM file examined in Integrated Genome Viewer (Broad Institute).

Every variant with a clonal size of, at least, 20% and >30 variant reads were bi-directionally sequenced using an ABI 3730 DNA Analyzer (Life Technologies, Carlsbad, CA, USA).

Six TP53-mutated cases were selected for applying the whole-amplicon panel on germline DNA (four cases from CD3+ sorted cells, two cases from buccal mucosal swab) to test its somatic nature.

### Statistical analysis

Comparisons of proportions and ranks of variables between groups were performed by *χ*^2^-test, Fisher's exact test, *t*-test or Mann–Whitney *U*-test, as appropriate. We used the Kaplan–Meier and the Cox method to analyse overall survival (OS) and progression-free survival, with a two-sided *P*-value⩽0.05 considered to be significant. In Cox models, examination of log (−log) survival plots and partial residuals was performed to assess that the underlying assumption of proportional hazards was met. Thresholds of <2.4 mg/dl for β2-microglobulin, an absolute B-cell count of 11 × 10^9^/l or over, and higher LDH levels than the upper normal limit (>UNL (that is, 378 U/l), were chosen as reported elsewhere.^23, 24, 25^

## Results

### Testing technique accuracy by resequencing

To estimate the accuracy of the technique to reproduce a variant call, 221 variants found in the first run were resequenced in depth (average reads per variant × 583). Eighteen of these variants had a clonal size over Sanger sensitivity (>20%), 58 variants with a clonal size between 10 and 20%, and 145 variants below 10% of clone size. For this experiment, new libraries were built to capture and amplify exclusively the amplicons covering the variants selected, and the same genomic DNA used in the first run was used. Receiver operating characteristic curves for both number of variant reads and variant allelic frequency (VAF) were created by plotting the true positive rate (sensitivity) against the false-positive rate (1-specificity) at various threshold settings. The number of variant reads in the initial run showed to be more accurate to predict the reproducibility of the variant in a second run, with an area under the curve of 0.894 (*P*⩽0.001; 95% CI, 0.817-0.970), than the clonal size (VAF) (area under the curve: 0.613; *P*=0.037; 95% CI, 0.487–0.738; Supplementary Figures 1A and 1B). A cutoff of 30 reads was chosen as threshold to consider a variant reproducible with a sensibility of 0.85 and a specificity of 0.945. No discrepancy was found when Sanger sequencing 15 selected variants over 20% of clonal size. None of the 13 variants harbored by six TP53-mutated cases was called when applying the panel on germline DNA.

#### Cohort and distribution of mutations

Two hundred and sixty-five patients were diagnosed in our center of a CLL from 2006 to 2012. Patients who, without needing therapy, did not achieve a minimum of 3 months of follow-up, were not contemplated (Figure 1). No monoclonal lymphocytosis cases were considered. The baseline characteristics of the 180 patients finally included were in accordance with their indolent-no need for treatment status at diagnosis, with 93% of the cohort assigned to Rai Stages 0 and I (Table 2).

**Figure 2:**
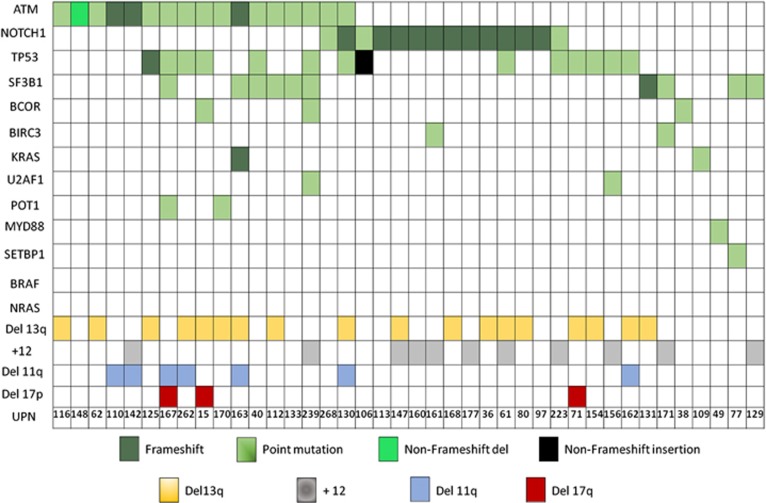
Distribution of mutations and chromosomal aberrations.

We found that 40/180 (22.2%) patients harbored at least one mutation; *ATM* (*n*=17, 9.4%), *NOTCH1* (*n*=14, 7.7%), *TP53* (*n*=14, 7.7%), *SF3B1* (*n*=10, 5.5%), *BCOR* (*n*=3, 1.6%), *BIRC3* (*n*=2, 1.1%), *KRAS* (*n*=2, 1.1%), *U2AF1* (*n*=2, 1.1%), *POT1* (*n*=2, 1.1%), *MYD88* (*n*=1, 0.6%), and *SETBP1* (*n*=1, 0.6%). No somatic variants were identified for *BRAF* and *NRAS* (Figure 2). Sixty-eight mutations were detected in the whole cohort with 18 deletions causing a frameshift, 1 non-frameshift deletion, 1 non-frameshift insertion and 48 missense single-nucleotide variants. Forty-one out of 68 mutations were already reported to the Catalog of Somatic Mutations in Cancer (COSMIC; http://cancer.sanger.ac.uk/cancergenome/projects/cosmic), as human cancers variants (Supplementary Table 1).

*SF3B1* and *NOTCH1* mutations were mutually exclusive, and a significant correlation between *NOTCH1* mutations and the presence of a trisomy 12 was found (*P*<0.01).

#### Clonal diversity of recurrently mutated genes in early-stage CLL

**Figure 3:**
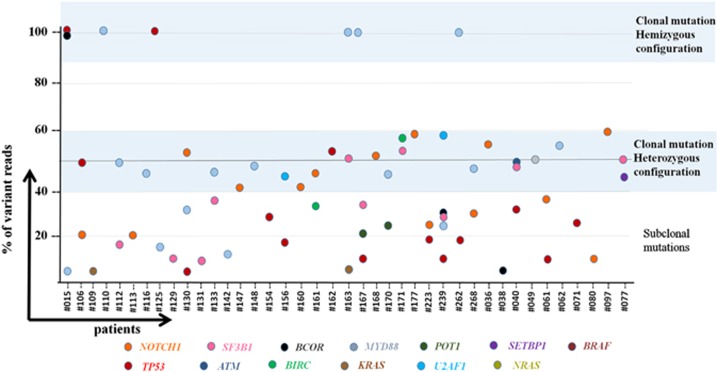
Estimated variant allele frequencies in the tumor fraction. The vertical axis represents allele frequency. Patients mutated are depicted in the abscissa. Color-coded circles for each gene affected. The variant allele frequency in non-sorted blood or bone marrow DNA was adjusted for the percentage of the immunophenotypically quantified CLL population.

As samples used for this sequencing study belong to the diagnostic immunophenotypic workout, we could estimate the clonal or subclonal nature of the acquired mutations, adjusting the variant allele burden in non-sorted blood or bone marrow DNA for the percentage of the immunophenotypically quantified CLL population. In eight patients with a del11q or del 17p and a *ATM* or *TP53* mutation, VAF was adjusted considering the loss of heterozygosity (Figure 3).

Thirty-five mutations were estimated to be clonal, that is, present in the whole tumor population, either in a heterozygous (*n*=28) or hemizygous (*n*=7) configuration. The allelic ratio of other mutations showed that they appeared only in a fraction of tumor cells, indicating that they were secondarily acquired or subclonal (*n*=33). Certain genes showed predominantly clonal mutations (71% of *NOTCH1*, 71% of *ATM* variants), whereas others were mainly subclonal (80% of *TP53* variants, 100% of Ras-family and *U2AF1* genes).

#### Clinical correlates

When confronting the 40 patients with, at least, one mutation, with the 140 non-mutated patients, we found less patients stratified as stage 0 in the mutated group, in favor of stages I and II. Focusing on FISH abnormalities, both 13q and +12 shown to be more frequent in mutated cases, though none of these differences reached the statistical significance (Table 2).

Likewise, no significant disparity was found when considering leukocyte, lymphocyte and platelet counts or hemoglobin, lactate dehydrogenase and β_2_-microglobulin levels. In addition, no specific CD38 or ZAP70 expression pattern was observed to be characteristic of each group.

Despite the low number of 17p and 11q deletions in our non-aggressive cohort, they were found mostly in the mutated group; the only difference statistically significant.

With a median follow-up of 54 months (interquartile range, 42–85 months), the median OS of the whole cohort has not been reached. Forty-two patients (23.3%) required therapy. Median time to treatment for all patients has not been reached. Considering only those patients who were treated, median time to first treatment was 48 months (range, 5–96 months).

**Figure 4:**
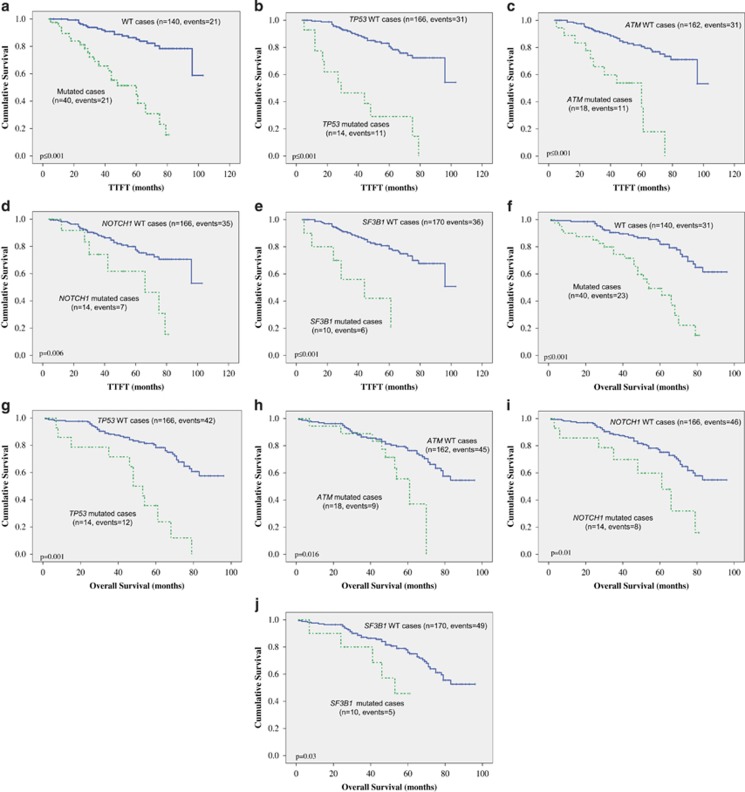
Differences in time to first treatment (TTFT) and survival outcomes (OS) in patients with, at least a mutation vs non-mutated (**a**, **f**); TP53 mutated or WT (**b**, **g**); ATM mutated or WT (**c**, **h**); NOTCH1 mutated or WT (**d**, **i**); and SF3B1 mutated or WT (**e**, **j**). *P*-values presented correspond to the Cox regression between the groups indicated.

Patients with, at least, one mutation had a worse time to first treatment (median TTFT: 60 months vs not reached; *P*⩽0.001; hazards ratio (HR)=5.8; 95% CI, 3.1–10.9; Figure 4a) than those cases without a detected mutation, and a shorter median OS (54 months vs not reached; *P*=0.01; HR=3.9; 95% CI, 2.2–6.9; Figure 4f).

TP53-mutated cases showed both a shorter TTFT (median 29 months vs not reached; *P*⩽0.001; HR=5.3; 95% CI, 2.6–10.8 CI; Figure 4b) and OS (median 48 months; *P*⩽0.001; HR=3.7; 95% CI, 1.9–7.2; Figure 4g) than the wild-type cases (median not reached). The presence of a mutation in *ATM* predicted both for a shorter TTFT (mean 60 months; *P*⩽0.001; HR=5; 95% CI, 2.5–10.2 CI; Figure 4c) and OS (median 61 months; *P*=0.016; HR=2.5; 95% CI, 1.2–5.2 CI; Figure 4h).

NOTCH1-mutated cases also presented a decreased TTFT (66 months; *P*=0.006; HR=3.1; 95% CI, 1.4–7.1; Figure 4d) and a shorter median OS (61 months; *P*=0.01; HR=2.7; 95% CI, 1.3–5.7; Figure 4i) than the wild-type cases (not reached). SF3B1-mutated patients also showed worse prognosis both in terms of TTFT (median 44 months; *P*⩽0.001; HR=5.3, 95% CI, 2.2-12; Figure 4e) and of OS (*P*=0.05; HR=2.7; 95% CI, 1–7.8; Figure 4j) than the wild-type cases (mean not reached).

The low number of del(11q) and del(17p) cases, and the potential correlation of regressors with *ATM* and *TP53* mutations (that is, variables strongly related to each other, measuring the same effect), precluded us from using them in this step of the analysis.

Mutational status of *TP53*, *ATM*, *NOTCH1*, *SF3B1*, *IgVH* status, the presence of +12, del(13q), the Rai Stage, the positive expression of CD38 and ZAP70, β2-microglobulin and LDH serum levels, and a B-cell count⩾11 × 10^9^/l were evaluated in a univariate Cox regression, both for TTFT and OS (Supplementary Tables 2A and B). Only those variables with a *P*⩽0.05 were included in a multivariate analysis (Tables 3A and B). The presence of a somatic variant in *TP53*, a positive CD38 antigen expression and β2-microglobulin serum levels above 2.4 mg/dl, prevailed as independent variables linked to a shorter time for needing treatment, whereas only *TP53* mutational status and β2-microglobulin levels remained as significant predictors for OS.

We next sought to explore the impact of *TP53* lesions below the sensitivity of Sanger. That is, to define the role of high-throughput sequencing in defining the prognosis in our cohort, as *TP53* mutations remained as the only independent genomic variable. In this sub-analysis we excluded five patients with a *TP53* lesion detectable by conventional techniques (direct sequencing and FISH/cytogenetics): one patient with a subclone harboring a *TP53* mutation and a del(17p), two patients with a clone with both a *TP53* and a del(17p), a case with a del(17p) in a clonal fashion but not detectable mutation, and one patient with a clonal *TP53* mutation and 37% of variant reads. The other 10 subclonal cases would have been missed by Sanger sequencing as they called in <20% reads (seven of them would have been missed even in B-cell-sorted DNA). We then replicated the multivariate Cox regression analyses shown in Tables 3A and B. Harboring one of these ten ‘sub-Sanger' *TP53* mutations granted an independent 3.5-fold increase of probability of needing treatment during the course of the disease than a wild-type patient (*P*=0.04; HR=3.5; 95% CI, 1–12.2), but it did not reach the significance for predicting OS (*P*=0.15; HR=2; 95% CI, 0.8–5.5).

Finally, a second sub-analysis showed that those patients with a double-hit *ATM* lesion (mutation+11q deletion) had the shorter median TTFT reported in this study (17 months) strikingly reduced compared with one-hit *ATM* patients (60 months). This impact was not seen in multivariate analysis or concerning OS (Supplementary Figure 2).

## Discussion

In the last few years a boost in the number of patients being diagnosed with early-stage CLL, not requiring therapy at diagnosis has been stated.^1, 2, 3^ In this subset, we have shown that *TP53* high-throughput mutational status emerges as an independent predictor, even when adjusting for the other recurrent gene variants in CLL, and for both traditional and recently reported prognostic factors.^24^ Surprisingly, even including newer genomic prognostic factors, a classical serologic parameter as β2-microglobulin levels, remained an independent predictor both for TTFT and OS, whereas a positive CD38 expression also independently predicted a shorter TTFT.

The baseline characteristics of the patients included in our study were in accordance with their indolent-no need for treatment status. As other groups have shown, the new definition of CLL, excluding those cases with a B-monoclonal population of <5000/ul, determined in our cohort a shift toward higher Rai Stages and a higher rate of patients progressing and needing therapy.^26, 27^

Patients with deletions on chromosome 17p respond worse to treatment than do those without it, resulting in early relapse and shorter survival.^28, 29, 30^ This cytogenetic lesion can be found in up to 50% of relapsed and refractory patients, but is rare at baseline (5–10%).^8^ We have found a 5% of *TP53* mutations in a subset of patients where a ‘wait and watch' therapeutical strategy is currently recommended. The relevant frequency and prognostic impact of *TP53* variants represented in <20% leukemic cells was recently reported by Rossi, *et al.*^13^ Of note, we show here that the majority of *TP53* mutations, at baseline in our inactive CLL subset, were under the sensitivity of conventional sequencing, and that they kept its independent impact on prognosis even when adjusting by high-throughput detected *ATM*, *NOTCH1* and *SF3B1* variants. Given the markedly short TTFT and OS of our TP53-mutated patients, it would seem an attractive approach to consider investigational studies directed to eradicate these subclones, in which these patients could be treated. Closely related, Farooqui *et al.*^31^ reported a remarkable 2-year survival and excellent drug-side-effects profile in the largest series of Ibrutinib therapy in treatment-naive patients with CLL and 17p deletions. In addition, identification of these subclones emerges as crucial as a concern is raised for clonal evolution and selection of resistant clones due the use of conventional (*p53*-dependent DNA damage) chemotherapeutic agents.^9, 32^

The frequency of *ATM* mutations in large series of CLL patients it is not so well understood, mostly owing to the fact that its size and the scattered distribution of its somatic mutations precludes the drafting of an amplicon-limited design.^33, 34^ We found *ATM* to be the most frequently mutated gene, with a predominance of clonal variants, indicating the ancestral-founding nature of these lesions. In addition, we found that the group with a shorter TTFT in our study was defined by those patients with a double-hit *ATM*. Likely, the reduced number of cases accounts for not reaching a statistical significance in multivariate or when addressing OS. Our finding supports the notion of a biological and clinical separation of double-hit *ATM* cases, from those where 11q deletion and the presence of an undamaged *ATM* allele give rise to a functional protein.^35, 36, 37^

Recent studies have suggested an important prognostic role for *NOTCH1* and *SF3B1* in CLL.^15, 38, 39^ However, no study has performed a multivariate analysis including traditional clinical and laboratory markers, flow cytometry factors, *IgVH* status and, at least, the presence of *ATM*, *TP53*, *NOTCH1* and *SF3B1* variants. In addition, using high-throughput sequencing seems essential to discern the independent prognostic value of these mutations, as sub-Sanger mutations accounted for 25% of the variants in our study. Though strongly associated with a worse outcome and TTFT in univariate analysis, *NOTCH1* and *SF3B1* did not kept its significance when including *TP53* and ATM mutations. Five out of seven, and four out of five *NOTCH1* and SF3B1-mutated cases, which needed therapy, also harbored a *TP53*/del 17p and/or *ATM*/del11q lesion. This co-occurrence with more aggressive alterations can explain, in part, the lack of independent predictive value of *SF3B1* and *NOTCH1* in our cohort. Consistent with previous reports including aggressive disease cases, in our early-stage CLL cohort, *SF3B1* and *NOTCH1* were mutually exclusive and a correlation between *NOTCH1* and trisomy 12 was found.^40, 41^

Previous studies have indicated that the prognostic significance of *IgVH* status is independent from that of classical clinical stages, markedly in patients with early-stage disease.^5, 42^ In our work, the inclusion of high-throughput mutational status ousted *IgVH* from the group of independent predictors, though it showed a trend for a shorter TTFT. ATM-mutated cases were found in a significant higher proportion among *IgVH*-unmutated cases. We did not find a different distribution of the *IgVH* status between TP53-mutated cases. These mutated cases can occur in both CLL *IgVH* subgroups. The late acquisition, autonomous of the emergence of the ancestral clone, of most *TP53* mutations (80% are subclones in our work), might justify this lack of association. Rossi *et al.*^13^ recently reported a similar frequency of *IgVH* non-mutated cases in *TP53* subclonal and wild-type cases.

Ours is a translational study, with the focus shifted to clinical pragmatism. In that sense, the clonal or subclonal nature of the mutations is not as crucial as its detectability by Sanger. Given that most of our cohort patients were lacking high-risk chromosomal alterations, we aimed to define the clinical impact of those variants with an allele frequency <20%. Of note, 10 out of 14 patients with a *TP53* lesion could be reported herein only by the use of next-generation sequencing. Most of *ATM* mutations were detectable by conventional PCR technique, but the extent and lack of hotspots in this gene makes capillary Sanger sequencing as time-consuming and labor-intensive as to preclude its use in the routine praxis. These two aspects strongly favor the use of targeted sequencing in routine CLL workout.

Surprisingly, β2-microglobulin levels, associated for the first time to CLL 25 years ago,^43^ showed its predictive value even compared with the newer biological markers of intrinsic cell phenotypic and genomic features. Of note, ours is not the first study to observe this strong role of β2-microglobulin in predicting outcome in this particular early-stage CLL subset.^23, 44^ The reason why a significant proportion of patients show high β2-microglobuline levels in the context of a low CLL burden disease still remains unclear. A positive expression of CD38 correlated with a shorter TTFT independently, a more expected finding, given that it showed an independent prognostic value (which ZAP70 and FISH findings failed to reach) in one of the largest study of prognostic markers carried out in Binet stage A patients with more than a thousand patients an 8 years of median follow-up.^42^

We acknowledge some caveats in our study. Considering FISH data as a loss of heterozygosity measure for VAF adjusting, can lead to underestimation of VAF in those cases where 17p or 11q clones might be under the sensibility threshold established for that probe. It is true that VAF would change, and *TP53* or *ATM* VAF estimates of <10% should be taken cautiously. However, those mutations would remain defined as subclonal, as none of our *TP53* or ATM-mutated patients had a CLL population of <25%. In addition, it could be argued we did not sequenced all *ATM* exons, and that we are underestimating *ATM* mutational status. Certainly, though we tried to design a cost-effective amplicon panel, and it covers most of *ATM* mutations reported in CLL, only an *ATM* whole-exome sequencing study will address the precise clinical impact of these gene variants. Finally, the lack of clinical grade availability of deep-sequencing data precludes its use presently. The implementation will need to go through standardization of methods and validation of prognostic value in clinical trials.

In sum, our finding of an independent prognostic value of *TP53* mutations, not detectable by conventional techniques in a subset of patients lacking treatment indication at baseline, adds another cobblestone to the positioning of amplicon deep-sequencing assays in established CLL diagnostics algorithms. The high-throughput determination of *TP53* status, particularly in this set of patients frequently lacking high-risk chromosomal aberrations, emerges as a key step, not only for prediction modeling, but also for exploring mutation-specific therapeutic approaches and minimal residual disease monitoring.

## Figures and Tables

**Table 1:** Targeted NGS panel characteristics

*Target gen*	*Length covered (bp)*	*% Gen exome sequenced*	% CLL mutations covered*	*Hotspot references*
*TP53*	2724	100%	100%	
*ATM*	7411	56%	78% (28 /36)	Wang *et al.,*^12^ Puente *et al.*^10^
*NOTCH1*	1487	19%	100% (75/75)	Wang *et al.,*^12^ Puente *et al.*^10^
*SF3B1*	3915	14%	92% (22/24)	Wang *et al.,*^12^ Puente *et al.*^10^
*MYD88*	296	31%	100% (11/11)	Wang *et al.,*^12^ Puente *et al.*^10^
*POT1*	549	28%	100% (5/5)	Wang *et al.,*^12^ Puente *et al.*^10^
*BIRC3*	963	53%	100% /(11/11)	Wang *et al.,*^12^ Puente *et al.*,^10^ Rossi *et al.*^45^
*KRAS*	567	100%	100%	
*NRAS*	570	100%	100%	
*U2AF1*	572	100%	100%	
*BRAF*	357	14%	100% (2/2)	Wang *et al.,*^12^ Puente *et al.*,^10^ Jebaraj *et al.*^46^
*BCOR*	5268	100%	100%	Wang *et al.,*^12^ Puente *et al.*^10^
*SETBP1*	3420	71%	100% (2/2)	Wang *et al.*,^12^ Puente *et al.*^10^
TOTAL	28099			

Abbreviations: bp, base pair; CLL, chronic lymphocytic leukemia. Genes included, total gene length covered, percentage of gene exon sequenced and percentage of CLL mutations covered using as reference those variants described in the whole-exome studies referred in the last column.

**Table 2:** Characteristics of patients included in the study at baseline and according to the presence or absence of, at least, a mutation

*Variable*	*Total (*n=*180)*	*(A) Mutated cases (*n=*40)*	*(B) Non-mutated cases (*n=*140)*	P *(A vs B)*
Age, years, (mean±s.d.)	69±1	70±12	68±11	0.9
Sex, male/female,% (*n*)	59 (107)/41 (74)	67 (27)/33 (13)	60 (85)/40 (55)	0.3
				
*Rai Stage, %*				
0	45	38	49	0.3
I	48	52	47	0.5
II	7	10	4	0.3
Leukocytes, x10^9^/l (median, IQR)	15.3 (10.9–26.9)	16.6 (10.9–24.2)	14.6 (10.7–24)	0.2
Lymphocytes, x10^9^/l (median, IQR)	10.3 (7–19.8)	12.2 (7.3–20.6)	9.8 (7–16)	0.3
B-cell count, x10^9^/l (median, IQR)	9.1 (6.1–17.3)	11.2 (7.4–19.1)	8.9 (6–15.6)	0.3
Hemoglobin, g/dl (median, IQR)	13.8 (12.2–14.7)	13.7 (12–14.5)	13.8 (12.3–14.7)	0.6
Platelets, x10^9^/l (median, IQR)	182 (136–217)	168 (126–216)	187 (136–217)	0.6
LDH IU/l (median, IQR)	368 (323–425)	377 (318–453)	324 (288–390)	0.4
B_2_microglobulin, mg/l (median, IQR)	2.2 (1.8–3)	2.4 (1.8–3.6)	2.2 (1.7–3)	0.3
ZAP70 +, % (*n*)	42 (76)	50 (20)	40 (56)	0.1
CD38 +, % (*n*)	28 (50)	38 (15)	22 (30)	0.1
IgHV-unmutated status, % (*n*)	34 (61)	43 (17)	27 (37)	0.07
				
*FISH abnormalities, % (*n*)*				
Del(13q)	46 (84)	50 (20)	47 (65)	0.7
+12	18 (32)	25 (10)	14 (20)	0.2
Del(11q)	5 (8)	17 (7)	1 (0.7)	**0.01**
Del(17p)	2 (4)	10 (4)	2 (1.4)	**0.02**

Abbreviations: CD38, cluster of differentiation 38; FISH, fluorescence in situ hibridization; IgVH, immunoglobulin heavy-chain variable region gen; IQR, interquartile range; LDH, lactate dehydrogenase; ZAP70, Zeta-chain-associated protein kinase 70; +12, trisomy 12. Bold face: del(11q) and del (17p) as variables distributed differently among mutated or not mutated patients with statistical significance (*P*=0.014)

.

Note: quantitative variables with a normal distribution are expressed as mean±s.d. Quantitative variables not following the normal distribution are expressed as median and interquartile ranges.

**Table 3A:** Multivariate Cox regression for time to first treatment

*Variable*	*RR (95% CI)*	P-*value*
**TP53**	**3.9 (1.4–10.9)**	**0.01**
IgVH	2 (0.9–4.4)	0.08
NOTCH1	1.9 (0.7–5.7)	0.2
ATM	1.9 (0.7–4.6)	0.17
+12	1.3 (0.6–2.7)	0.4
**CD38**	**2.3 (1–5.3)**	**0.04**
SF3B1	2.6 (0.8–8)	0.09
Rai Stage>0	1.1 (0.5–2.3)	0.7
B-cell count ⩾10 × 10^9^/l	1.9 (0.9–3.9)	0.08
β**2-microglobulin** ⩾**2.4**	**2.4 (1.2–4.8)**	**0.02**

Abbreviations: CD38, cluster of differentiation 38; CI, confidence interval; IgVH, immunoglobulin heavy-chain variable region gen; RR, relative risk; +12, trisomy 12. Bold face: variables reaching the independent statistical significance.

**Table 3B:** Overall survival

*Variable*	*RR (95% CI)*	P-*value*
**TP53**	**2.5 (1.2–6.4)**	**0.02**
IgVH	1.5 (0.8–2.6)	0.2
β**2-microglobulin** ⩾**2.4**	**1.9 (1.1–3.4)**	**0.04**
NOTCH1	1.8 (0.7–4.5)	0.2
SF3B1	2.5 (0.9–7.2)	0.08
Rai Stage >0	1.1 (0.5–2.3)	0.7
ATM	0.9 (0.4–2.5)	0.9

Abbreviations: CD38, cluster of differentiation 38; CI, confidence interval; IgVH, immunoglobulin heavy-chain variable region gen; RR, relative risk; +12, trisomy 12. Bold face: variables reaching the independent statistical significance.
