# Supplementary material for: Prognostic signature and clonality pattern of recurrently mutated genes in inactive chronic lymphocytic leukemia
Source: Blood Cancer J. 2015 Aug 28;5(8):e342–. doi: 10.1038/bcj.2015.65 (PMC4558590; doi:10.1038/bcj.2015.65)
Supplement: Supplementary Information [file bcj201565x2.doc]

**Supplementary Material**

**Supplementary Table 2. Univariate Cox regression for TTFT (left) and OS (right).**

**
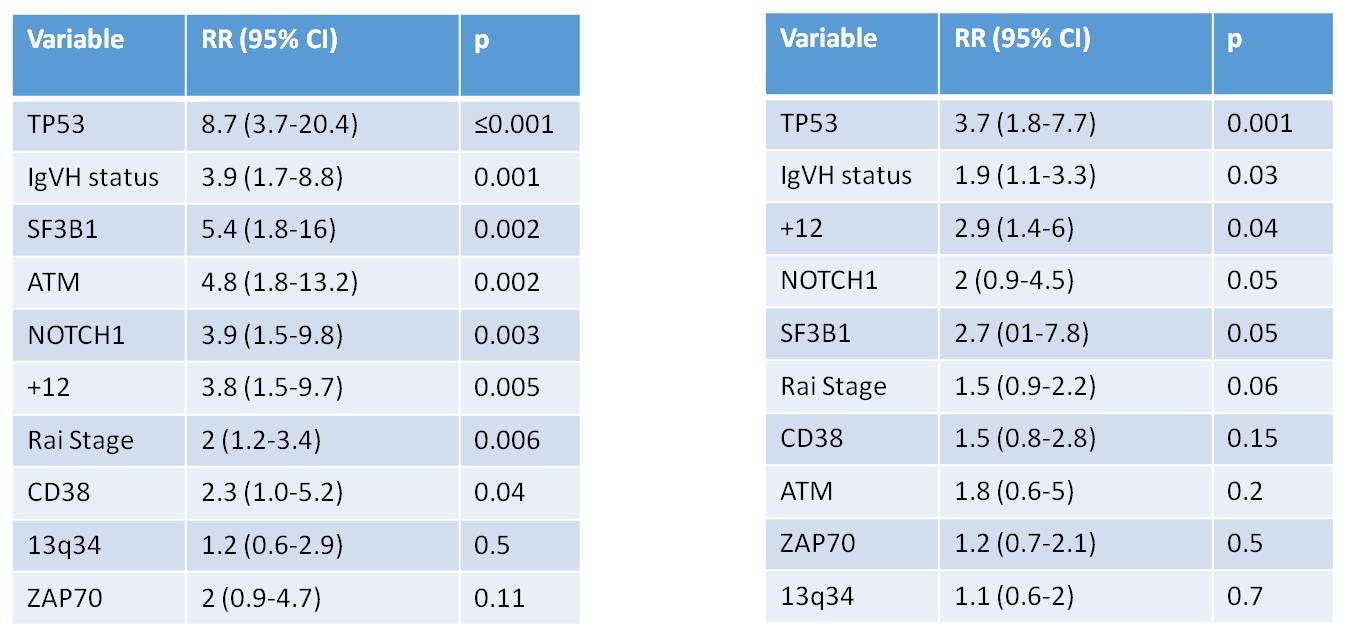
**

**Supplementary Figure 1. Receiver operating characteristic (ROC) curves illustrating the performance of predicting the reproducibility of a 1^st^ run mutation in the resequencing run, according to: A) number of variant reads (AUC 0.894); B) variant allele frequency (AUC 0.613).**

**
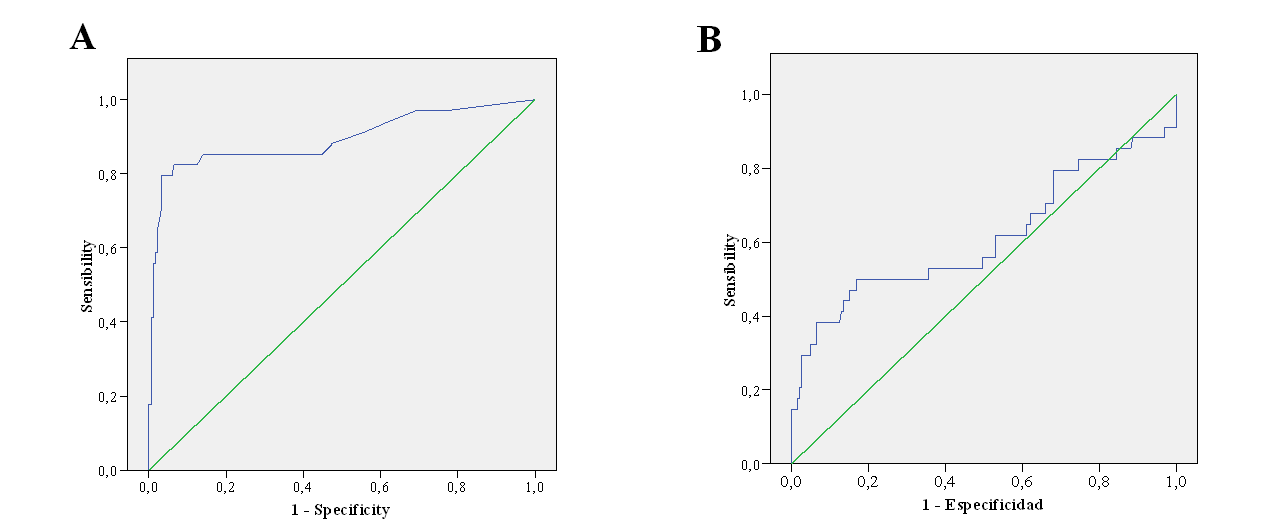
**

**Supplementary Figure 2. Differences in time to first treatment (TTFT) (right) and survival outcomes (OS) (left) in patients with, double-hit vs. one-hit ATM**P values presented correspond to the Cox regression between the groups indicated.

**
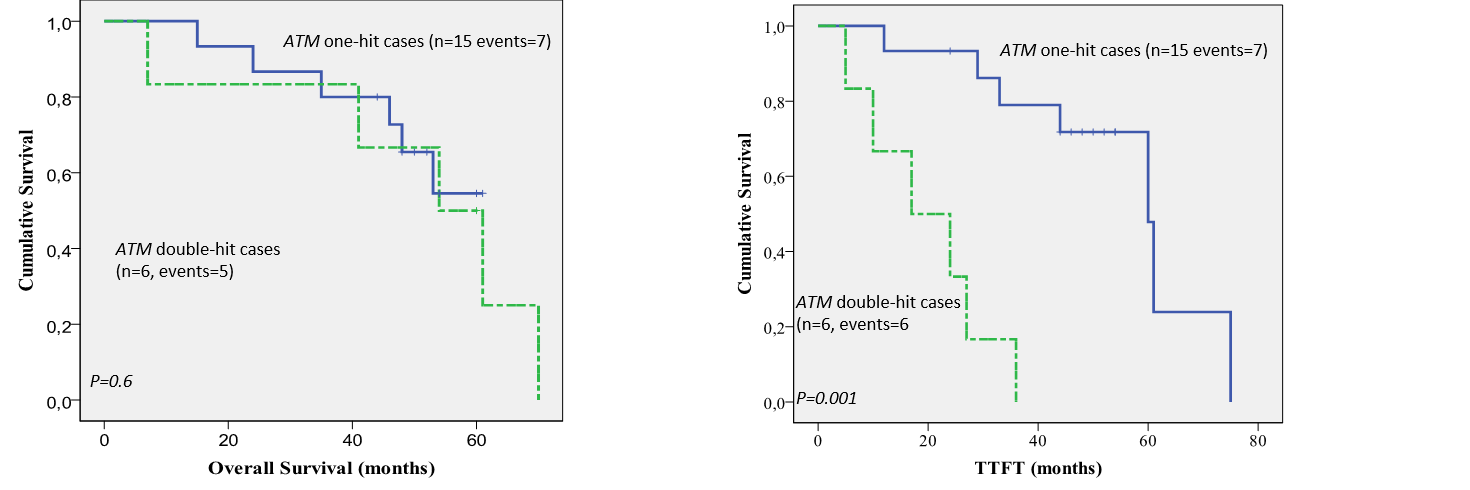
**
